# Genome-Wide Analysis of DNA Methylation Dynamics during Early Human Development

**DOI:** 10.1371/journal.pgen.1004868

**Published:** 2014-12-11

**Authors:** Hiroaki Okae, Hatsune Chiba, Hitoshi Hiura, Hirotaka Hamada, Akiko Sato, Takafumi Utsunomiya, Hiroyuki Kikuchi, Hiroaki Yoshida, Atsushi Tanaka, Mikita Suyama, Takahiro Arima

**Affiliations:** 1 Department of Informative Genetics, Environment and Genome Research Center, Tohoku University Graduate School of Medicine, Sendai, Japan; 2 JST, CREST, Saitama, Japan; 3 St. Luke Clinic Laboratory, Oita, Japan; 4 Yoshida Ladies Clinic Center for Reproductive Medicine, Sendai, Japan; 5 St. Mother Clinic Laboratory, Kitakyushu, Fukuoka, Japan; 6 Division of Bioinformatics, Medical Institute of Bioregulation, Kyushu University, Maidashi 3-1-1, Fukuoka, Japan; King's College London, United Kingdom

## Abstract

DNA methylation is globally reprogrammed during mammalian preimplantation development, which is critical for normal development. Recent reduced representation bisulfite sequencing (RRBS) studies suggest that the methylome dynamics are essentially conserved between human and mouse early embryos. RRBS is known to cover 5–10% of all genomic CpGs, favoring those contained within CpG-rich regions. To obtain an unbiased and more complete representation of the methylome during early human development, we performed whole genome bisulfite sequencing of human gametes and blastocysts that covered>70% of all genomic CpGs. We found that the maternal genome was demethylated to a much lesser extent in human blastocysts than in mouse blastocysts, which could contribute to an increased number of imprinted differentially methylated regions in the human genome. Global demethylation of the paternal genome was confirmed, but SINE-VNTR-Alu elements and some other tandem repeat-containing regions were found to be specifically protected from this global demethylation. Furthermore, centromeric satellite repeats were hypermethylated in human oocytes but not in mouse oocytes, which might be explained by differential expression of *de novo* DNA methyltransferases. These data highlight both conserved and species-specific regulation of DNA methylation during early mammalian development. Our work provides further information critical for understanding the epigenetic processes underlying differentiation and pluripotency during early human development.

## Author Summary

DNA methylation reprogramming after fertilization is critical for normal mammalian development. Early embryos are sensitive to environmental stresses and a number of reports have pointed out the increased risk of DNA methylation errors associated with assisted reproduction technologies. Therefore, it is very important to understand normal DNA methylation patterns during early human development. Recent reduced representation bisulfite sequencing studies reported partial methylomes of human gametes and early embryos. To provide a more comprehensive view of DNA methylation dynamics during early human development, we report on whole genome bisulfite sequencing of human gametes and blastocysts. We show that the paternal genome is globally demethylated in blastocysts whereas the maternal genome is demethylated to a much lesser extent. We also reveal unique regulation of imprinted differentially methylated regions, gene bodies and repeat sequences during early human development. Our high-resolution methylome maps are essential to understand epigenetic reprogramming by human oocytes and will aid in the preimplantation epigenetic diagnosis of human embryos.

## Introduction

In mammals, DNA methylation is essential for normal development and plays critical roles in repression of transposable elements, maintaining genome stability, genomic imprinting and X-chromosome inactivation. DNA methylation patterns are relatively stable in somatic cells but genome-wide reprogramming of DNA methylation occurs in primordial germ cells and preimplantation embryos [1]–[3]. During mouse preimplantation development, the maternal genome is passively demethylated in a replication-dependent manner while some oocyte-specific methylated regions maintain maternal allele-specific methylation at the blastocyst stage [4], [5]. In contrast, the paternal genome is actively and rapidly demethylated through the oxidation of 5-methylcytosine (5mC) to 5-hydroxymethylcytosine (5hmC) by ten-eleven translocation-3 [6]. In spite of the global demethylation, imprinted differentially methylated regions (DMRs) and some transposable elements (*e.g.* intracisternal A-particles (IAPs)) are specifically protected from demethylation [1].

During human preimplantation development, the paternal genome is reported to be actively demethylated as in the mouse [7], [8], but the regulatory mechanism and the genome-wide DNA methylation patterns in early embryos are not well understood. Recently, two studies employed reduced representation bisulfite sequencing (RRBS) of human gametes and early embryos to characterize the human methylome very early in development [7], [9]. According to these studies, the paternal genome is rapidly and globally demethylated after fertilization whereas demethylation of the maternal genome is more limited and some oocyte-specific methylated regions maintain monoallelic methylation during preimplantation development, similar to the mouse genome. RRBS is known to cover 5–10% of genomic CpGs, favoring those contained within CpG islands (CGIs) and promoter regions. To obtain an unbiased and more complete representation of the methylome during early human development, we performed whole genome bisulfite sequencing (WGBS) of human gametes and blastocysts that covered>70% of genomic CpGs. We found human-specific regulation of DNA methylation in various regions including oocyte-methylated CGIs, gene bodies and tandem repeat-containing regions.

## Results

### WGBS of human gametes and blastocysts

We performed WGBS of human oocytes, sperm, blastocysts and neonatal blood cells. For ethical reasons, we used only surplus germinal vesicle (GV) or metaphase I (MI) oocytes and blastocysts obtained from female patients undergoing *in vitro* fertilization (IVF) treatment. Sperm and blood cells were collected from healthy donors (see Materials and Methods for details). WGBS libraries were constructed using the amplification-free post-bisulfite adaptor tagging (PBAT) method [10] for all samples except the oocytes, which required PCR-amplification (PCR cycles  = 10) to increase the read depth (Table 1). For each cell type, 87–96% of genomic CpGs were covered by at least one read, which was comparable to the reported methylome maps of mouse gametes [5], [11], [12] and human sperm [13]. We also compared two oocyte PBAT libraries prepared with and without PCR-amplification (Oocyte(+PCR) and Oocyte(−PCR)) (S1A Figure, S1B Figure, and Table 1). The methylation levels of individual CpGs were highly correlated (*r* = 0.83) between these two libraries. Furthermore, the average methylation levels were very similar: Oocyte(+PCR) at 53.1% versus Oocyte(−PCR) at 54.8%. These data demonstrate that our PCR-amplification protocol did not lead to significant bias in our data sets. Non-CpG methylation was observed in human oocytes, especially at CpA sites (mean  = 5.6%), with a positive correlation between CpG and non-CpG methylation (S1C Figure, S1D Figure). Non-CpG methylation was not a significant feature of sperm or blastocysts (<1%). In the following analyses, only CpGs covered with ≥3 reads were considered for oocytes and those covered with ≥5 reads were considered for the other samples.

**Table 1:** Summary of whole genome bisulfite sequencing.

Sample	Number	Mapped reads	Depth	≥1	≥3	≥5
Oocyte (+PCR)	79 oocytes	144,463,623	4.5	74.4%	56.9%	38.5%
Oocyte (−PCR)	123 oocytes	79,772,565	2.5	71.9%	26.9%	6.6%
Oocyte (Total)	202 oocytes	224,236,188	7.0	87.5%	71.0%	54.1%
Sperm (Donor-1)	-	243,284,702	7.6	90.7%	80.1%	68.5%
Sperm (Donor-2)	-	258,580,093	8.1	91.2%	80.8%	68.9%
Sperm (Donor-3)	-	280,502,462	8.8	91.3%	82.0%	71.8%
Sperm (Total)	3 individuals	782,367,257	24.5	94.9%	91.3%	88.2%
Blastocyst	80 embryos	750,044,631	23.5	95.7%	93.6%	91.3%
Blood (Donor-1)	-	102,369,166	3.2	81.4%	46.8%	21.8%
Blood (Donor-2)	-	107,690,372	3.4	84.0%	53.3%	26.7%
Blood (Donor-3)	-	98,147,071	3.1	83.4%	51.0%	24.1%
Blood (Donor-4)	-	100,978,105	3.2	83.5%	51.3%	24.9%
Blood (Donor-5)	-	113,611,080	3.6	76.8%	35.5%	11.4%
Blood (Total)	5 individuals	522,795,794	16.4	94.7%	90.4%	85.4%

Libraries were prepared without PCR-amplification except for Oocyte (+PCR). The proportion (%) of CpGs covered with over 1, 3 or 5 reads is indicated. Oocyte (Total), Sperm (Total) and Blood (Total) are used in most our analyses. Bisulfite conversion rates were>99% for all samples.

We confirmed that three imprinted DMRs and two pluripotency genes frequently observed to be abnormal in poor quality oocytes or embryos [14], [15] were normally methylated in our WGBS data (S1E Figure). We also compared our WGBS data with recently reported RRBS data of human oocytes, blastocysts and inner cell mass (ICM) and WGBS data of ICM [7], [9]. Our data substantially increased the coverage of genomic CpGs compared with the reported data (S1F Figure, S1G Figure). The methylation levels of CGIs showed high correlations (*r* = 0.96) between our WGBS data and the reported RRBS data (oocyte: S1H Figure, blastocyst: S1I Figure), validating the WGBS data.

### Global changes of DNA methylation during early human development

**Figure 1:**
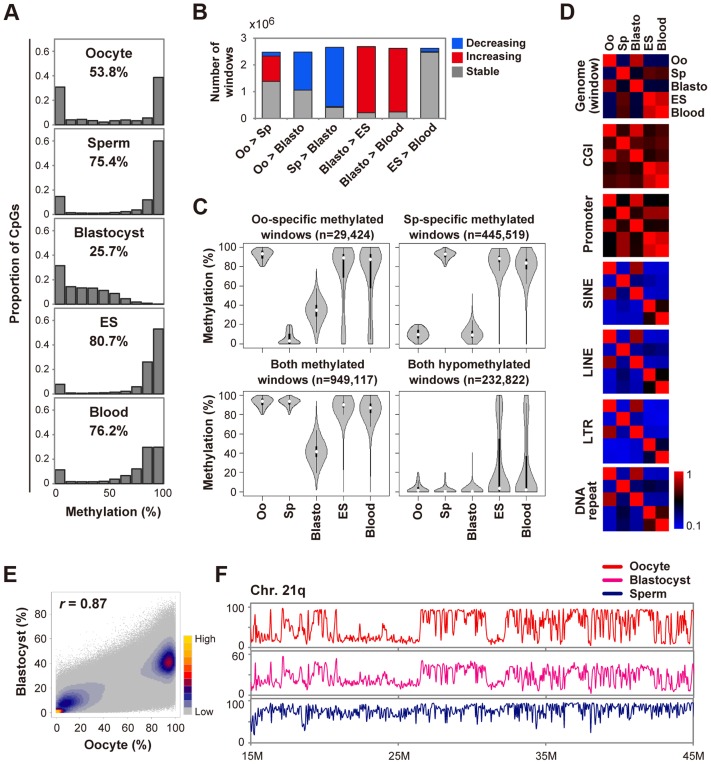
Global changes of DNA methylation during early human development. **A,** Distribution of methylation levels of individual CpGs. The mean methylation levels of CpGs are also indicated. We included human H9 ES cells (GEO accession number: GSM706059) for comparison. **B,** Detection of dynamic methylation changes using a sliding window (window size  = 20 CpGs, step size  = 10 CpGs). Windows were classified as increasing (or decreasing) if the methylation levels increased (or decreased) by>20% and the changes were significant (BH-corrected *P*<0.05). The other windows were classified as stable. Oo: Oocyte; Sp: Sperm; Blasto: Blastocyst. **C,** Violin plots of mean methylation levels of windows hypermethylated (≥80%) or hypomethylated (≤20%) in one or both gametes. Oo-specific (Sp-specific) methylated windows are defined as windows hypermethylated in oocytes (sperm) and hypomethylated in sperm (oocytes). Thin and thick lines are box plots and white dots indicate the median. **D,** Heatmaps of Pearson correlation coefficients. Correlation coefficients were calculated based on the mean methylation levels of individual windows, CGIs, promoters and repeat copies. Correlation coefficients are color-coded as shown. **E,** A density scatterplot of mean methylation levels of the sliding windows. The Pearson correlation coefficient between oocytes and blastocysts was high (*r* = 0.87). The density is color-coded as indicated. **F,** Methylation levels across the long arm of chromosome 21 (smoothed using 50 kb non-overlapping windows). Similar methylation patterns were observed for oocytes and blastocysts whereas the methylation levels of blastocysts were low (note that the vertical maximum scale is 60% for blastocysts).

Similar to findings for the mouse, human oocytes showed an intermediate methylation level of CpGs and blastocysts were globally hypomethylated (Fig. 1A). To further characterize global DNA methylation changes, we used a system of sliding windows of 20 CpGs with a step size change of 10 CpGs. Windows were classified as increasing (or decreasing) if the methylation levels increased (or decreased) by>20% and the changes were statistically significant (Benjamini-Hochberg (BH) corrected *P*<0.05). We found that 57% and 83% of windows showed decreased methylation levels in blastocysts compared with oocytes and sperm, respectively (Fig. 1B). In contrast,>90% of windows showed increased methylation in ES or blood cells compared with blastocysts (Fig. 1B). To explore the differences in demethylation dynamics between parental genomes, we focused on windows hypermethylated in one gamete and hypomethylated in the other. In this study, we defined regions that were ≥80% methylated as hypermethylated and those that were ≤20% methylated as hypomethylated. Windows hypermethylated in sperm and hypomethylated in oocytes (sperm-specific methylated windows) were abundant in intergenic regions. In contrast, oocyte-specific ones showed a relatively uniform distribution (S2A Figure). In blastocysts, oocyte-specific methylated windows showed intermediate methylation levels (median  = 35.1%), in contrast to the nearly complete demethylation of sperm-specific ones (Fig. 1C). Almost all windows hypomethylated in both gametes remained hypomethylated and very few windows (0.04%) were hypermethylated in blastocysts, suggesting that genome-wide *de novo* methylation occurred after implantation (Fig. 1C and S2B Figure). Consistently, the methylation patterns of oocytes and blastocysts were very similar to each other (Figs. 1E, F), suggesting that the global methylation pattern of the maternal genome, but not the paternal genome, was inherited by blastocysts.

Next, we examined specific genomic features: CGIs, promoters and transposable elements. CGIs and promoters hypermethylated in sperm remained methylated in ES and blood cells. On the other hand, oocyte-specific methylated CGIs showed variable methylation levels and oocyte-specific methylated promoters were preferentially demethylated in ES and blood cells (S3A Figure and S3B Figure). In addition, the promoter methylation patterns of sperm, but not of oocytes, showed high correlations with those of ES and blood cells (*r*>0.8, Fig. 1D). These data highlighted the unique promoter methylation profile of oocytes. Short interspersed nuclear elements (SINEs), long interspersed nuclear elements (LINEs), long terminal repeats (LTRs) and DNA repeats were essentially highly methylated in ES and blood cells, whereas 20–30% and 3–8% of repeat copies were hypomethylated in oocytes and sperm, respectively (S2B Figure). These transposable elements were demethylated similarly to other genomic regions in blastocysts (S3 Figure).

### Stability of imprinted DMRs and oocyte-specific methylated CGIs

**Figure 2:**
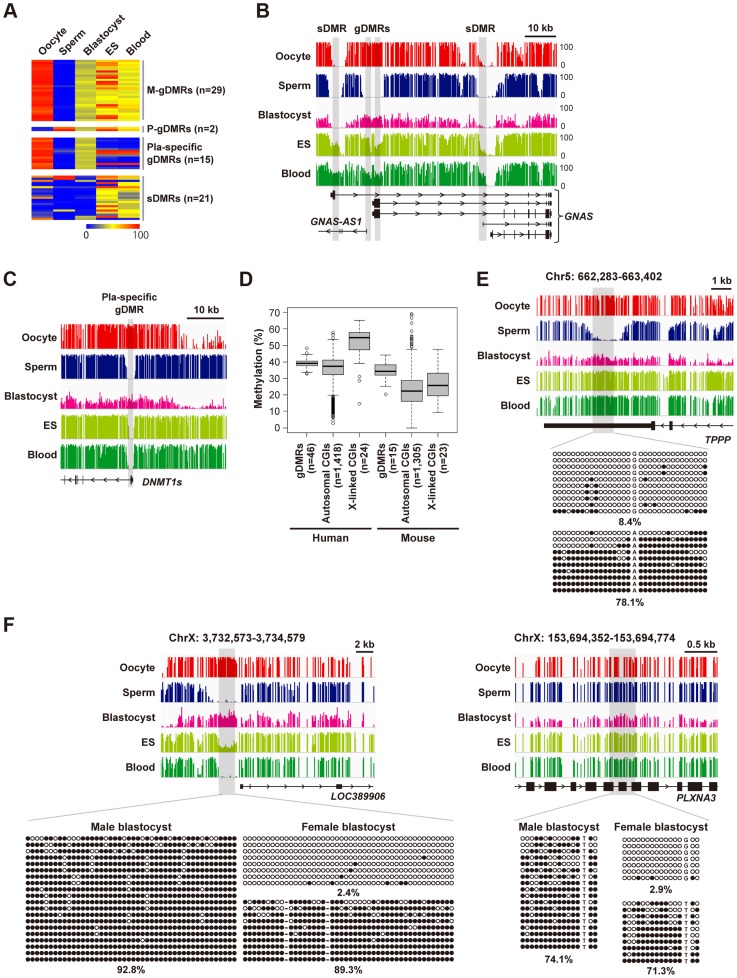
Establishment and maintenance of imprinted DMRs. **A,** A heatmap of mean methylation levels of imprinted DMRs. We classified the 67 known human imprinted DMRs [17], and found that 44 were maternal germline DMRs (M-gDMRs), 2 were paternal germline DMRs (P-gDMRs) and 21 were secondary DMRs (sDMRs). 15 M-gDMRs are reported to be maintained only in the placenta and shown as “Pla-specific gDMRs”. gDMRs other than placenta-specific ones showed 35–65% methylation levels in blood cells but the intermediate methylation levels were not well maintained in ES cells (11/31 showed>75% methylation). Methylation levels are color coded as indicated. The raw data are shown in S1 Table. **B,** Methylation patterns at the human *GNAS* locus. The vertical axis indicates the methylation level (%). In this locus, there were two gDMRs and two sDMRs. All DMRs overlap promoter regions. **C,** Methylation patterns at the human *DNMT1* locus. The promoter region of the somatic isoform of *DNMT1* (*DNMT1s*) is known to show maternal allele-specific methylation in the placenta [45]. The *DNMT1* DMR was hypomethylated in both ES and blood cells, suggesting placenta-specific protection of the maternal allele from demethylation. **D,** Box plots of mean methylation levels of gDMRs and oocyte-specific methylated CGIs in blastocysts. Boxes represent lower and upper quartiles and horizontal lines indicate the median. Whiskers extend to the most extreme data points within 1.5 times the interquartile range from the boxes. The open circles indicate the data points outside the whiskers. Methylation levels of mouse gDMRs and oocyte-specific methylated CGIs [5] are shown for comparison. **E,** Methylation patterns of an oocyte-specific methylated CGI. A single blastocyst was used for the analysis. Black and white circles indicate methylated and unmethylated residues, respectively. The percentages of methylated CpG sites are indicated. **F**, Bisulfite sequencing analyses of X-linked CGIs hypermethylated in oocytes. A single blastocyst was used for each bisulfite sequencing analysis.

Germline DMRs (gDMRs) frequently serve as imprinting control regions [16] and we were interested in how many gDMRs exist in the human genome. Among the 67 known imprinted DMRs [17], 46 DMRs were classified as gDMRs according to the following definition: DMRs hypermethylated in one gamete and hypomethylated in the other (Fig. 2A, B and S1 Table). Of these, 15 reportedly placenta-specific DMRs were lost in blood cells (Fig. 2A, C). The other 31 gDMRs showed intermediate methylation levels in blood cells, but about one-third of these gDMRs were not maintained in ES cells (H9 ES cells: Fig. 2A, H1 and HUES6 ES cells: S4A Figure), indicating the instability of gDMRs in human ES cells. Importantly, oocyte-specific methylated autosomal CGIs showed methylation levels very similar (median  = 37.5%) to gDMRs (median  = 39.2%) in human blastocysts (Fig. 2D). We confirmed monoallelic methylation of four autosomal CGIs in human blastocysts by using conventional bisulfite sequencing (Fig. 2E and S4B Figure). We also analyzed two X-linked CGIs hypermethylated in oocytes and found that these CGIs showed high methylation levels in male blastocysts (the X chromosome of male blastocysts is derived from oocytes) and monoallelic methylation in female blastocysts (Fig. 2F). Consistently, X-linked CGIs with oocyte-specific methylation showed higher methylation levels than autosomal ones in blastocysts (the WGBS data were derived from a pool of blastocysts) (Fig. 2D). A similar tendency was also observed in the sliding window-based analyses (S2C Figure). These data suggested that a substantial number of oocyte-specific methylated CGIs may maintain maternal allele-specific methylation in human blastocysts. In contrast, most oocyte-specific methylated CGIs were significantly demethylated compared with gDMRs in mouse blastocysts (Fig. 2D).

### A bimodal gene body methylation pattern associated with transcription in human oocytes

**Figure 3:**
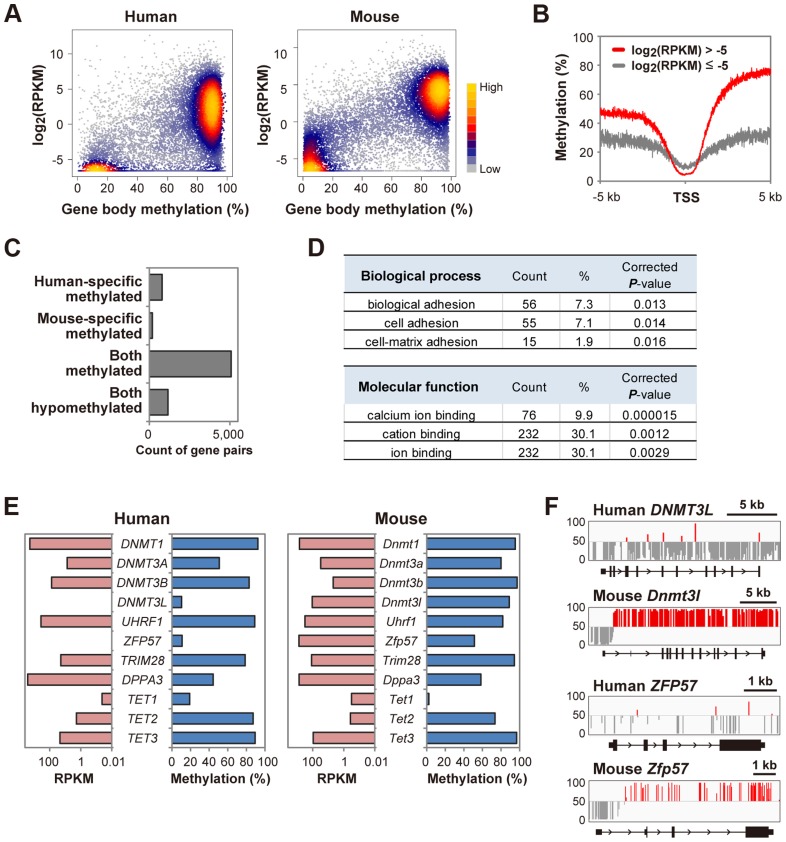
A bimodal gene body methylation pattern associated with transcription in human oocytes. **A,** A density scatterplot of gene body methylation levels and transcription levels [43] in human oocytes. The data of mouse oocytes [5], [11] are also shown for comparison. Only genes longer than 5 kb were analyzed. For genes with RPKM less than 0.01, RPKM was set as 0.01. The density is color-coded as indicated. **B,** Mean methylation levels within 5 kb of transcription start sites (TSS) in human oocytes. Genes (>5 kb) were classified into two groups (log_2_(RPKM)>−5 and ≤−5). Methylation levels were smoothed using 5 bp non-overlapping sliding windows. **C,** Conservation of gene body methylation levels between human and mouse oocytes. 783 and 188 genes showed human-specific and mouse-specific gene body hypermethylation, respectively. 5076 and 1151 genes were hypermethylated and hypomethylated in both types of oocytes, respectively. The raw data are shown in S2 Table. **D,** GO analysis of 783 genes with human-specific gene body hypermethylation. The top three GO terms (biological process and molecular function) are indicated with gene counts, the proportion (%) and BH-corrected *P*-values. No GO term was enriched in genes with mouse-specific gene body hypermethylation. **E,** Gene body methylation levels and transcription levels of DNA methylation regulators in human and mouse oocytes. *DNMT3L* and *ZFP57* showed gene body hypomethylation and were not expressed (RPKM<0.01) in human oocytes. *DNMT3B* (RPKM = 76.0) showed 10-fold higher expression than *DNMT3A* (RPKM = 7.6) in human oocytes. In contrast, *Dnmt3b* (RPKM = 4.9) showed ∼6-fold lower expression than *Dnmt3a* (RPKM = 30.6) in mouse oocytes. **F,** Methylation patterns at human *DNMT3L* and *ZFP57* loci and mouse *Dnmt3l* and *Zfp57* loci. The vertical line indicates the methylation level (%) and the baseline is set at 50% to highlight unmethylated CpGs. CpGs with>50% and <50% methylation are shown in red and grey, respectively.

In mouse oocytes, gene-body methylation levels are reported to positively correlate with the transcription levels [5]. In human oocytes, a positive correlation between gene-body methylation and transcription levels was also observed. Interestingly, there was an expression-level boundary at around log_2_(RPKM)  = −5 (RPKM: reads per kilobase per million) (Fig. 3A). Genes with log_2_(RPKM)>−5 and <−5 may be transcriptionally active and inactive genes, respectively (Fig. 3B). We analyzed previously reported mouse methylome and transcriptome data and found that a bimodal distribution of gene body methylation was also observed while there was a boundary at around log_2_(RPKM)  = 0 (Fig. 3A). It is unclear whether the difference between the human and mouse expression-level boundaries reflects experimental or functional differences. We found that 971 genes showed differential gene body methylation between human and mouse oocytes (Fig. 3C and S2 Table). Gene ontology (GO) analysis revealed an abundance of genes encoding cell adhesion molecules with human-specific gene body hypermethylation (Fig. 3D), which could have important roles during human oogenesis. In mouse oocytes, *Dnmt3l* and *Zfp57* are highly expressed and essential for DNA methylation regulation [18], [19] whereas human *DNMT3L* is undetectable in oocytes [20]. Here we found that the gene body regions of *DNMT3L* and *ZFP57* were hypomethylated in human oocytes and neither gene was expressed (Figs. 3E, F), implying that *DNMT3L* and *ZFP57* might not be essential for regulation of DNA methylation in human oocytes.

### Unique regulations of tandem repeat-containing regions

**Figure 4:**
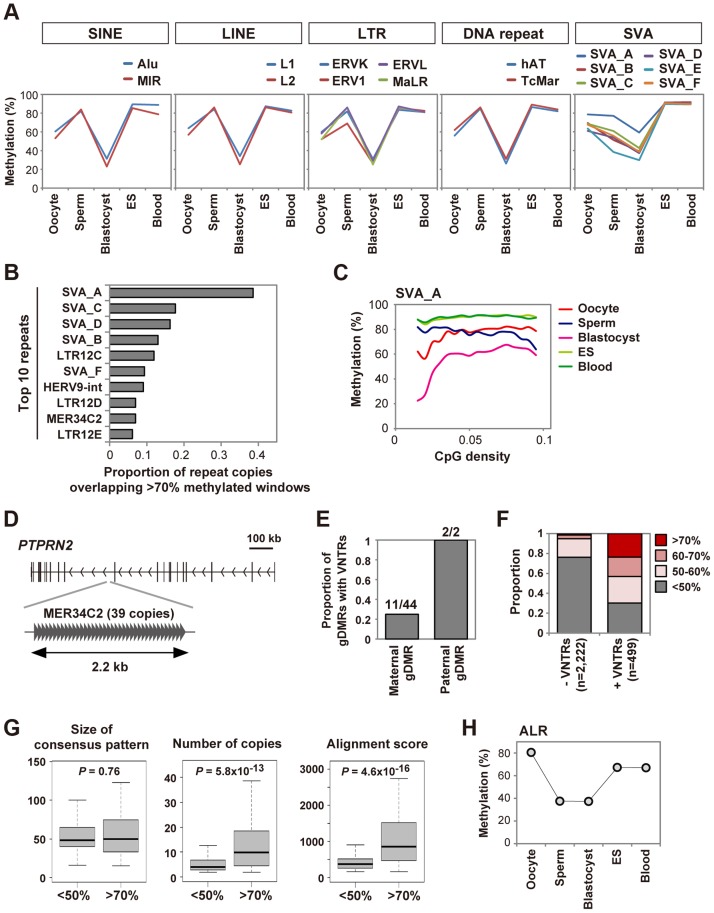
Unique regulation of tandem repeat-containing regions. **A,** DNA methylation dynamics of transposable elements. Mean methylation levels of CpGs in various classes of SINEs, LINEs, LTRs and DNA repeats and SVA subfamilies are shown. SVA_A showed an especially high methylation level in blastocysts (59.2%). **B,** Proportions of repeat copies overlapping>70% methylated windows in human blastocysts. We analyzed only SINEs, LINEs, LTRs, DNA repeats, SVAs and satellites with>100 copies in the human genome. The top ten repeat names with the highest proportions are shown. The raw data are shown in S4 Table. **C**, Relationships between methylation levels and CpG densities. Mean methylation levels of CpGs in SVA_A are plotted against CpG densities. **D,** MER34C2 copies overlapping>70% methylated windows in human blastocysts. 39 MER34C2 copies are all tandemly repeated within the *PTPRN2* gene locus. **E,** Proportions of maternal and paternal gDMRs containing VNTRs. Counts of gDMRs with VNTRs and total gDMRs are indicated. **F,** Proportions of mean methylation levels of CGIs with and without VNTRs in human blastocysts. Only autosomal CGIs hypermethylated in both gametes were analyzed. 118 of 499 CGIs with VNTRs and 31 of 2,222 CGIs without VNTRs showed>70% methylation (*P* = 0, chi-square test). **G,** Characteristics of VNTRs highly methylated in blastocysts. Using Tandem Repeats Finder [41], the size of the consensus pattern, the number of tandemly aligned copies and the alignment score were compared between VNTRs of <50% methylated CGIs and>70% methylated CGIs shown in (F). The alignment score calculated by Tandem Repeat Finder reflects the degree of similarity between repeat copies. When several VNTRs were found in a CGI, the VNTR with the highest alignment score was analyzed. Boxes represent lower and upper quartiles and horizontal lines indicate the median. Whiskers extend to the most extreme data points within 1.5 times the interquartile range from the boxes. The Mann-Whitney U test was used to calculate *P*-values. No sequence motif was found among the consensus patterns of the>70% methylated CGIs using DREME [42]. **H,** Mean methylation levels of CpGs in ALR. Oocytes showed the highest methylation level (80.6%).

As described above, global methylation changes of SINEs, LINEs, LTRs and DNA repeats were very similar to other genomic regions in early human embryos (S3 Figure). We further analyzed mean methylation levels of CpGs in various classes of these transposable elements (Fig. 4A, see also S3 Table for details). These repeat classes showed similar methylation changes: ∼60% methylated in oocytes, ∼80% methylated in sperm, ES and blood cells and ∼30% methylated in blastocysts. These data suggested that SINEs, LINEs, LTRs and DNA repeats were essentially not resistant to genome-wide demethylation after fertilization. Mouse IAPs are known to be protected from demethylation during preimplantation development [5], [21]. To identify transposable elements specifically protected from demethylation during human preimplantation development, we screened repeat copies overlapping windows showing>70% methylation in blastocysts (0.3% of all windows) (S4 Table). We found that SINE-VNTR-Alu (SVA) subfamilies, especially SVA_A, frequently overlapped the>70% methylated windows (Fig. 4B). SVA_A also showed the highest methylation level in blastocysts (59.2%) whereas the other repeat sequences were <50% methylated (Fig. 4A and S3 Table). SVA is a hominid-specific repeat family that remains active in the human genome [22]. Similar to mouse LTRs [5], methylation levels of CpGs within SVAs are positively correlated with CpG density in human oocytes and blastocysts (Fig. 4C and S5 Figure). LTR12 subfamilies, which are LTRs of HERV9, also tended to overlap the>70% methylated windows (Fig. 4B). Interestingly, both SVA and LTR12 subfamilies contain CpG-rich variable number tandem repeats (VNTRs) [22], [23]. We also noticed that whereas the MER34C2 consensus sequence does not contain VNTRs, MER34C2 copies overlapping the>70% methylated windows were all tandemly repeated in a single genomic locus (Fig. 4D). VNTRs were also found in the two paternal gDMRs (Fig. 4E). VNTRs were not a common feature of the maternal gDMRs, but a significantly higher proportion of the maternal gDMRs did contain VNTRs as compared with all CGIs (gDMRs: 11/44, CGIs: 1763/27718, chi-square *P* = 4.1×10^−7^). Therefore, we focused on CGIs hypermethylated in both gametes and found that CGIs containing VNTRs were preferentially protected from demethylation in blastocysts (Fig. 4F). A comparison between VNTRs of>70% and <50% methylated CGIs in blastocysts revealed that VNTRs with more repeats tended to be protected from demethylation, whereas no sequence motif was found (Fig. 4G). These data suggested that VNTRs might underlie silencing of specific transposable elements and the protection of paternal gDMRs.

We also found that alpha satellite (ALR), which is a tandemly repeated DNA family found in centromeric and pericentromeric regions [24], was hypermethylated in human oocytes (80.6%) (Fig. 4H). Interestingly, *DNMT3B* was highly expressed in human oocytes (Fig. 3E), and DNMT3B is reported to interact with centromere protein CENP-C and contribute to DNA methylation of ALR [25]. Thus, it is possible that DNMT3B is involved in DNA methylation of ALR in human oocytes.

## Discussion

This work reports the genome-wide DNA methylation patterns of human gametes and blastocysts at single-base resolution. Our WGBS data of oocytes and blastocysts substantially increase the coverage of genomic CpGs adding to the reported RRBS data of oocytes and blastocysts and WGBS data of ICM [7], [9]. We confirmed that the paternal genome was globally demethylated as previously reported. However, the oocyte-specific methylated regions maintained intermediate methylation levels in human blastocysts (median  = 35.1%). Consistently, the methylation patterns of oocytes and blastocysts were very similar to each other, suggesting that the global methylation pattern of the maternal genome was inherited by blastocysts. Furthermore, oocyte-specific methylated CGIs showed methylation levels very similar (median  = 37.5%) to gDMRs (median  = 39.2%). These data appear not to support replication-dependent global demethylation of the maternal genome during human early development, because oocyte-specific methylated regions should show ≤25% methylation after one replication-dependent global demethylation event. In mouse blastocysts, most oocyte-specific methylated CGIs were significantly demethylated compared with gDMRs, which may reflect the passive demethylation of the maternal genome [1], [2]. These data strongly suggest that the maternal genome is demethylated to a much lesser extent in human blastocysts than in mouse blastocysts.

We classified known imprinted DMRs [17] and discovered that there were at least 46 gDMRs in the human genome including 15 specific to the placenta. Our data suggested that a substantial number of oocyte-specific methylated CGIs may also maintain mono-allelic methylation in human blastocysts whereas they were essentially lost through hypermethylation or hypomethyaltion in blood cells. It is suggested that a significant portion of gene transcripts show mono-allelic expression in human 8-cell embryos and morulae [26], and the oocyte-specific methylated CGIs could regulate mono-allelic expression of some genes in human preimplantation embryos. In the mouse genome, ∼25 well defined gDMRs have been identified and only the *Gpr1* DMR is reported to be placenta-specific [27], [28]. The demethylation resistance of oocyte-specific methylated CGIs during early human development may, in part, explain the increased number of placenta-specific gDMRs in the human genome. Interestingly, we found that *ZFP57* was not expressed in human oocytes. Because replication-dependent global demethylation of the maternal genome is not likely to occur during human preimplantation development, we speculate that the protection of gDMRs by *ZFP57* may be dispensable in human oocytes. These data contribute to our understanding of the regulatory mechanism of human-specific genomic imprinting.

Both human and mouse oocytes showed bimodal gene body methylation patterns associated with transcription. While it is unclear whether transcription is the only determinant, transcription may be an important determinant of the oocyte methylomes. In mammals, DNMT3A and DNMT3B are *de novo* DNA methyltransferases whereas DNMT3L acts in a recruiting role. In mouse oocytes, *Dnmt3a* and *Dnmt3l* are essential for *de novo* DNA methylation, whereas *Dnmt3b* is poorly expressed and essentially dispensable [11], [29]. In contrast, in human oocytes *DNMT3B* showed ∼10-fold higher expression than *DNMT3A*, and *DNMT3L* was not expressed, suggesting that *DNMT3B* may be the critical *de novo* DNA methyltransferase during human oocyte growth. Interestingly, centromeric satellite repeats were highly methylated in human oocytes. These regions are known to be hypomethylated in mouse oocytes [30]. Human DNMT3B is reported to interact with centromere protein CENP-C and contribute to DNA methylation of centromeric satellite repeats [25]. Similarly, centromeric satellite repeats are demethylated in *Dnmt3b* mutant mice [31]. Therefore, the differential expression pattern of DNMT3B could explain this human-specific hypermethylation of centromeric satellite repeats in oocytes.

It is suggested that evolutionarily young SINEs and LINEs are demethylated to a milder extent than older ones during human preimplantation development [7]. We found that SVAs and some LTRs containing CpG-rich VNTRs were much more preferentially protected from demethylation than SINEs and LINEs in human blastocysts. Paternal gDMRs also contained VNTRs and many VNTR-containing CGIs remained highly methylated in human blastocysts. Therefore, VNTRs might underlie the protection of paternal gDMRs and specific transposable elements from demethylation. The maintenance of DNA methylation of SVAs may be especially important because SVAs are currently active in the human genome and are involved in various human diseases [22], [32]. While the underlying mechanism of the protection of VNTR-containing regions is currently unknown, it is noteworthy that VNTRs are related to RNA-directed DNA methylation in plants [33]. Many transposable elements including SVAs are expressed in human early embryos [7], [9] and it is interesting to speculate that RNA might be involved in the demethylation resistance of VNTR-containing regions.

Overall, this work highlights both conserved and species-specific regulation of DNA methylation during early mammalian development. Our WGBS data of human gametes and blastocysts not only provide information to support our understanding of normal human developmental processes but also will be useful in interpreting studies on assisted reproductive technologies (ARTs). ARTs in humans are associated with an increased risk of imprinting disorders [34], [35], and our data will aid in the safety evaluation of ARTs and the preimplantation epigenetic diagnosis of human embryos.

## Materials and Methods

### Sample collection

Human oocytes, sperm, blastocysts and umbilical cord blood cells were obtained with signed informed consent of the donors or the couples, and the approval of the Ethics Committee of Tohoku University School of Medicine (Research license 2013-1-57), associated hospitals, the Japan Society of Obstetrics and Gynecology and the Ministry of Education, Culture, Sports, Science and Technology (Japan). Altogether, 202 surplus oocytes and 80 surplus blastocysts were obtained from female patients (ages 26–43) undergoing IVF treatment. The patients were healthy women with no habitual drug use and no particular past or familial disease history. We collected morphologically normal GV and MI oocytes from preovulatory follicles by intravaginal ultrasound-guided follicular aspiration after controlled ovarian hyperstimulation. To remove cumulus cells and the zona pellucida, oocytes were treated with hyaluronidase solution (JX Nippon Oil & Energy Corporation, Tokyo, Japan) and Tyrode's solution-Acidified (JX Nippon Oil & Energy Corporation) according to the manufacturer's instructions. Blastocysts were obtained by culturing early cleavage-stage embryos in Global Medium (LifeGlobal, Guilford, CT) overlaid with mineral oil. We used morphologically normal expanding or expanded blastocysts. The number of ICM cells is similar to, or a little lower than, that of trophectoderm (TE) cells in blastocysts at this stage [36]. Because ICM and TE cells show similar methylation levels [7], [9] and the available embryos in this study were limited, we performed WGBS using whole blastocysts. Ejaculated sperm samples with normal volume, counting and rates of mortality were collected. Only motile sperm cells isolated by the swim-up method [37] were used.

### Construction and sequencing of PBAT libraries

Oocytes and blastocysts were incubated in a lysis solution (0.1% SDS, 1 mg/ml proteinase K, 50 ng/µl carrier RNA (QIAGEN, Valencia, CA)) for 60 min at 37°C and then 15 min at 98°C. Genomic DNA was purified with phenol/chloroform extraction and ethanol precipitation. Sperm genomic DNA was prepared as described [38]. Genomic DNA of cord blood cells was purified with phenol/chloroform extraction and ethanol precipitation. Isolated genomic DNA was spiked with 5% (for oocytes and blastocysts) or 0.5% (for sperm and cord blood cells) unmethylated lambda DNA (Promega, Madison, WI). Bisulfite treatment was performed using the MethylCode Bisulfite Conversion Kit (Invitrogen, Carlsbad, CA).

PBAT libraries were prepared as previously described [10]. Briefly, the first-strand DNA was synthesized with the Klenow fragment (3′-5′ exo-) (NEB, Beverly, MA) using BioPEA2N4 (5′-biotin-ACA CTC TTT CCC TAC ACG ACG CTC TTC CGA TCT NNN N-3′). The biotinylated first-strand DNA was captured using Dynabeads M-280 Streptavidin (Invitrogen). The second-strand DNA was synthesized with the Klenow fragment (3′-5′ exo-) using PE-reverse-N4 (5′-CAA GCA GAA GAC GGC ATA CGA GAT NNN N-3′). After removing the first-strand DNA, the second strand was double stranded with Phusion Hot Start II High-Fidelity DNA Polymerase (Finnzymes, Woburn, MA) using Primer-3 (5′-AAT GAT ACG GCG ACC ACC GAG ATC TAC ACT CTT TCC CTA CAC GAC GCT CTT CCG ATC T-3′). For an oocyte PBAT library, PCR-amplification was performed with KAPA HiFi HotStart Uracil+ ReadyMix (2×) (Kapa Biosystems, Woburn, MA) using primers, (5′-CAA GCA GAA GAC GGC ATA CGA GAT-3′) and (5′- AAT GAT ACG GCG ACC ACC GAG ATC T-3′). The following program was used for the PCR-amplification: 10 cycles of 98°C for 15 sec, 65°C for 30 sec and 72°C for 30 sec. Concentrations of the PBAT libraries were measured by quantitative PCR (qPCR) using the Kapa Library Quantification Kit (Kapa Biosystems).

PBAT libraries were sequenced on the HiSeq 2000 or HiSeq 2500 platform (Illumina, CA, USA) with 100-bp single-end reads using the TruSeq SR Cluster Kit v3-cBot-HS and the TruSeq SBS Kit v3-HS (Illumina).

### Mapping and methylation analysis

Sequenced reads were processed using the Illumina standard base-calling pipeline (v1.8.2) and the first 4 bases were trimmed to remove random primer sequences. The resulting reads were aligned to the reference genome (UCSC hg19) using Bismark [39] (v.0.9.0) with default parameters. For the oocyte library prepared with PCR-amplification, identical reads were treated as a single read to remove PCR duplicates. The methylation level of each cytosine was calculated using the Bismark methylation extractor. For CpG sites, reads from both strands were combined to calculate the methylation levels. Except for S1 Figure, methylation levels of CpGs covered with ≥3 reads were analyzed for oocytes and those of CpGs covered with ≥5 reads were analyzed for the other samples. Bisulfite conversion rates were estimated using reads that uniquely aligned to the lambda phage genome and were>99% for all samples. In this study, mC and hmC were indistinguishable because bisulfite sequencing cannot differentiate hmC from mC.

We also included available RRBS data of human oocytes, ICM and blastocysts [7], [9] and WGBS data of human ICM [7], ES cells (H1, H9 and HUES6) and mouse oocytes [11]. Processed methylation data were downloaded from NCBI GEO (http://www.ncbi.nlm.nih.gov/geo) for ICM (Accession number: GSE49828 and GSE51239), blastocysts (Accession number: GSE51239), H1 (Accession number: GSM429321), H9 (Accession number: GSM706059) and HUES6 ES cells (Accession number: GSM1173778). The RRBS data from biological replicates were combined. For mouse oocytes [11], the raw reads were mapped to the reference genome (UCSC mm9) and analyzed as described above (only CpGs covered with ≥5 reads were used).

### Annotations of genomic regions

Annotations of Refseq genes, CGIs and repeat sequences were downloaded from the UCSC Genome Browser. Refseq genes shorter than 300 bp (encoding microRNAs or small nucleolar RNAs in most cases) were excluded from our analyses. Promoters were defined as regions 1 kb upstream and downstream from transcription start sites of Refseq transcripts. For calculation of the mean methylation levels, we analyzed only CGIs and promoters containing ≥10 CpGs with sufficient coverage for calculation of the methylation levels. Similarly, we considered only repeat copies containing ≥5 CpGs for calculation of the mean methylation levels of repeat copies. The gene bodies were defined as transcribed regions of Refseq transcripts except for promoters. When several Refseq transcripts were assigned to a Refseq gene, the transcribed regions were merged into a single gene body. Regions and names of the 67 imprinted DMRs were defined as previously reported [17].

The CpG density was defined for each CpG site as the density of CpGs within 100 bp upstream and downstream regions (the number of CpGs was divided by 200). Gene ontology analyses were performed using the Database for Annotation, Visualization and Integrated Discovery (DAVID) [40]. The list of human and mouse homologs including HomoloGene IDs was downloaded from Mouse Genome Informatics (MGI, http://www.informatics.jax.org/). VNTRs were identified using Tandem Repeats Finder [41] (alignment parameters  = 2, 5, 7; minimum alignment score  = 150; maximum period size  = 500). Sequence motifs among VNTRs were searched using DREME [42] (the consensus patterns of VNTRs of>70% and <50% methylated CGIs in Fig. 4G were used as positive and negative sequences, respectively).

### Transcriptome analysis

Transcriptome data of human and mouse oocytes were previously reported [5], [43]. The raw reads from biological replicates were combined and analyzed using Avadis NGS software with default parameters (version 1.5, Strand Scientific Intelligence).

### Graphical presentation

Methylation levels of CpGs were visualized using Integrative Genomics Viewer (IGV) software (http://www.broadinstitute.org/igv/). Heatmaps and scatter plots were generated using the heatmap.2 function of the gplots package and the heatscatter function of the LSD package in R (http://www.R-project.org/), respectively. Violin plots were generated using the vioplot package (http://neoscientists.org/~plex/).

### Sliding window-based analysis of methylation changes

We used a sliding window of 20 CpGs with a step size of 10 CpGs (the mean length was ∼2 kb) for consideration of the successful identification of imprinted DMRs using sliding windows of 10 CpGs [44] and 25 CpGs [17]. We considered only windows containing ≥10 CpGs with sufficient coverage for calculation of the methylation levels (84% of windows were covered in all samples shown in Fig. 1). Windows were classified as increasing (or decreasing) if the methylation levels increased (or decreased) by>20% and the changes were statistically significant according to Student's *t*-test with BH correction (*P*<0.05).

### Bisulfite sequencing

DNA samples were treated with sodium bisulfite using an EZ DNA Methylation Kit (Zymo Research, Orange, CA) and PCR-amplified using TaKaRa EpiTaq™ HS (Takara Bio, Shiga, Japan). The PCR products were cloned into the pGEM-T Easy vector (Promega) and individual clones were sequenced. The following primers were used: chr5: 662,283–663,402: (5′-GGG GTT AAG ATG GGA GTT ATG A-3′) and (5′-TAA ACA ACC CAA TCC CCA CA-3′), chr12: 20,704,525-20,706,004: (5′-GGG AGG AGG AGG AGT AGT AGG A-3′) and (5′-CCC ACT AAA AAC AAA ATC AAT ACC-3′), chr15: 89,952,271-89,953,061: (5′-GAT TTT TGT TAA TGA TTG GGT AGG A-3′) and (5′-CCC CAC AAT ATC TAC CCT CAT A-3′), chr21: 32,716,044-32,716,485: (5′-AGA AGT TAA GGG GGA AAG ATG A-3′) and (5′-TTC ACA AAT TAC ACC CAC TAC CTC-3′′), chrX: 3,732,573-3,734,579: (5′-TTA ATG GGG TAA AGG GGT TAG A-3′) and (5′-ACC AAA TAA ACC CCA CCC AAA C-3′), chrX: 153,694,352-153,694,774: (5′-GTG GGG TTT AAG GAA GGA GGT A-3′) and (5′-CAA TCA CCC ACA CAC AAC TCC-3′). The sex of blastocysts was determined by PCR amplification of the male-specific SRY locus using bisulfite-converted DNA with the following primers: Forward: (5′ -TGA AAT TAA ATA TAA GAA AGT GAG GGT TG- 3′) and Reverse: (5′ -CCA CAC ACT CAA AAA TAA AAC ACC A- 3′).

### Accession number

All sequencing data are deposited in the Japanese Genotype-phenotype Archive under the accession number JGAS00000000006.

## Supporting Information

S1 Figure **Summary of whole genome bisulfite sequencing.** **A,** Mean methylation levels of cytosines in oocytes. Methylation levels of individual cytosines covered with at least one read were analyzed. PCR amplification did not affect overall methylation levels of cytosines. H = A, T or C. **B,** Pearson correlation coefficients between replicates. Methylation levels of individual CpGs covered with at least 3 reads were used for the calculation. Correlation coefficients were high (>0.70) in all cases. **C,** Mean methylation levels of individual non-CpG sites. Non-CpG sites covered with at least one read were analyzed. **D,** A density scatterplot of CpG and non-CpG methylation levels of oocytes. The methylation levels were calculated with a non-overlapping sliding window of 10 kb. Cytosines covered with at least one read were analyzed. The density is color-coded as indicated. **E,** Mean methylation levels of imprinted DMRs (*KvDMR1*, *MEST* and *H19*) and the promoters of pluripotency genes (*POU5F1* and *NANOG*). The *KvDMR1* and *MEST* DMR were hypermethylated and the *H19* DMR, *POU5F1* and *NANOG* were hypomethylated in oocytes. In blastocysts, imprinted DMRs showed intermediate methylation levels but the pluripotency genes were hypomethylated. These patterns are frequently disrupted in poor-quality oocytes or preimplantation embryos derived from patients undergoing ART [14], [15]. **F**, Proportions of CpGs covered by the oocyte WGBS data from this study and RRBS data [7]. Only CpGs covered with ≥3 reads were considered. **G,** Proportions of CpGs covered by the blastocyst WGBS data of this study and previously reported blastocyst/ICM WGBS or RRBS data [7], [9]. Only CpGs covered with ≥5 reads were considered. **H,** A density scatterplot of mean methylation levels of CGIs in oocytes. A high correlation was observed between our WGBS data and reported RRBS data [7]. The density is color-coded as indicated. **I,** A density scatterplot of mean methylation levels of CGIs in blastocysts. A high correlation was observed between our WGBS data and reported RRBS data [9].
(TIF)

S2 Figure **DNA methylation levels of specific genomic regions.** **A,** Genomic distribution of windows. The proportions of windows overlapping promoters, exons, introns and intergenic regions are indicated. If a window overlaps more than two categories, the priority is as follows: 1) promoter, 2) exon, 3) intron, 4) intergenic region (*e.g.* if a window overlaps a promoter and an exon, it is classified as “promoter”). Sperm-specific methylated windows were abundant in intergenic regions. More than half of the windows hypomethylated in both gametes overlapped promoters. **B,** Distribution of mean methylation levels of windows, CGIs, promoters and repeat copies. A high proportion of hypomethylated repeat copies is evident in oocytes and blastocysts. **C,** Box plots of mean methylation levels of the sliding windows in human blastocysts. Boxes represent lower and upper quartiles and horizontal lines indicate the median. Whiskers extend to the most extreme data points within 1.5 times the interquartile range from the boxes. X-linked windows hypermethylated in oocytes showed ∼10% higher methylation levels than autosomal ones.
(TIF)

S3 Figure **Region-specific methylation changes during early human development.** **A,** Violin plots of mean methylation levels of CGIs. Thin and thick lines are box plots and white dots indicate the median. **B,** Violin plots of mean methylation levels of promoters. Oocyte-specific methylated promoters preferentially showed low methylation levels in ES and blood cells. **C–F,** Violin plots of mean methylation levels of repeat copies. SINEs, LINEs, LTRs and DNA repeats were demethylated similarly to other genomic regions in blastocysts.
(TIF)

S4 Figure **Stability of gDMRs and oocyte-specific methylated CGIs.** **A,** A heatmap of mean methylation levels of gDMRs in H1 (GEO accession number: GSM429321) and HUES6 (GEO accession number: GSM1173778) ES cells. Among gDMRs other than placenta-specific ones, 13 and 9 DMRs showed>75% methylation in H1 and HUES6 ES cells, respectively. Methylation levels are color-coded as indicated. **B**, Methylation patterns of three oocyte-specific methylated CGIs. Black and white circles indicate methylated and unmethylated residues, respectively. The percentages of methylated CpG sites are indicated.
(TIF)

S5 Figure **Relationships between methylation levels and CpG densities.** Mean methylation levels of CpGs in six repeat families are plotted against CpG densities. All genomic CpGs were also analyzed for comparison. Mean methylation levels were calculated only for CpG densities with>1000 CpG sites covered by all samples. Transposable elements were essentially highly methylated in ES and blood cells. Low methylation levels of CpGs were observed in oocytes and blastocysts regardless of the CpG density. In sperm, CpGs in SINEs, LTRs and satellites showed especially low methylation levels at high CpG densities.
(TIF)

S1 Table **DNA methylation levels of imprinted DMRs.** DMRs are classified into four groups: Maternal gDMRs; oocyte-specific methylated gDMRs with 35–65% methylation levels in blood cells, Paternal gDMRs; sperm-specific methylated gDMRs with 35–65% methylation levels in blood cells, Placenta-specific maternal gDMRs; maternal gDMRs maintained only in the placenta, Secondary DMRs; DMRs other than gDMRs. For secondary DMRs, neighboring gDMRs are indicated. While the *ZC3H12C* and *LIN28B* DMRs are classified as secondary DMRs, these may be placenta-specific maternal gDMRs (the methylation levels in oocytes were 76.0% and 77.4%, respectively).
(XLSX)

S2 Table **Gene body methylation and transcription levels in human and mouse oocytes.** Only genes longer than 5 kb were analyzed. Homologous genes between the human and mouse are shown with HomoloGene IDs.
(XLSX)

S3 Table **DNA methylation levels of repeat sequences.** We analyzed SINEs, LINEs, LTRs, DNA repeats, SVAs and satellites with>100 copies in the human genome. Mean methylation levels of CpGs are shown.
(XLSX)

S4 Table **Proportion of repeat copies highly methylated in human blastocysts.** We analyzed SINEs, LINEs, LTRs, DNA repeats, SVAs and satellites with>100 copies in the human genome. Proportions of repeat copies overlapping>70% methylated windows are indicated.
(XLSX)
