# Supplementary figures and images for: Genome-Wide Analysis of DNA Methylation Dynamics during Early Human Development
Source: PLoS Genet. 2014 Dec 11;10(12):e1004868. doi: 10.1371/journal.pgen.1004868 (PMC4263407; doi:10.1371/journal.pgen.1004868)

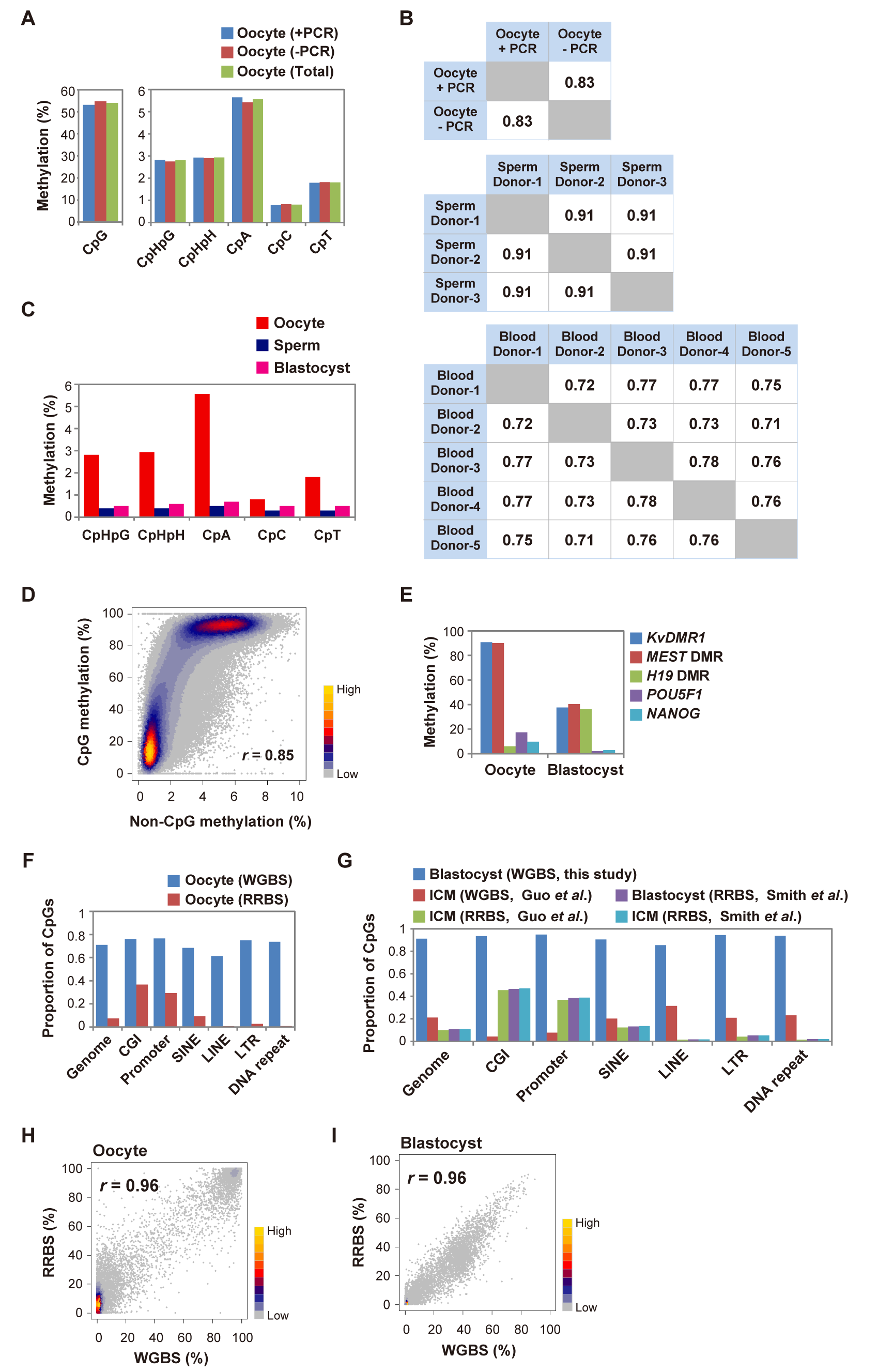

Supplement: S1 Figure — Summary of whole genome bisulfite sequencing. A, Mean methylation levels of cytosines in oocytes. Methylation levels of individual cytosines covered with at least one read were analyzed. PCR amplification did not affect overall methylation levels of cytosines. H = A, T or C. B, Pearson correlation coefficients between replicates. Methylation levels of individual CpGs covered with at least 3 reads were used for the calculation. Correlation coefficients were high (>0.70) in all cases. C, Mean methylation levels of individual non-CpG sites. Non-CpG sites covered with at least one read were analyzed. D, A density scatterplot of CpG and non-CpG methylation levels of oocytes. The methylation levels were calculated with a non-overlapping sliding window of 10 kb. Cytosines covered with at least one read were analyzed. The density is color-coded as indicated. E, Mean methylation levels of imprinted DMRs (KvDMR1, MEST and H19) and the promoters of pluripotency genes (POU5F1 and NANOG). The KvDMR1 and MEST DMR were hypermethylated and the H19 DMR, POU5F1 and NANOG were hypomethylated in oocytes. In blastocysts, imprinted DMRs showed intermediate methylation levels but the pluripotency genes were hypomethylated. These patterns are frequently disrupted in poor-quality oocytes or preimplantation embryos derived from patients undergoing ART [14], [15]. F, Proportions of CpGs covered by the oocyte WGBS data from this study and RRBS data [7]. Only CpGs covered with ≥3 reads were considered. G, Proportions of CpGs covered by the blastocyst WGBS data of this study and previously reported blastocyst/ICM WGBS or RRBS data [7], [9]. Only CpGs covered with ≥5 reads were considered. H, A density scatterplot of mean methylation levels of CGIs in oocytes. A high correlation was observed between our WGBS data and reported RRBS data [7]. The density is color-coded as indicated. I, A density scatterplot of mean methylation levels of CGIs in blastocysts. A high correlation was observed between o [file pgen.1004868.s001.tif]

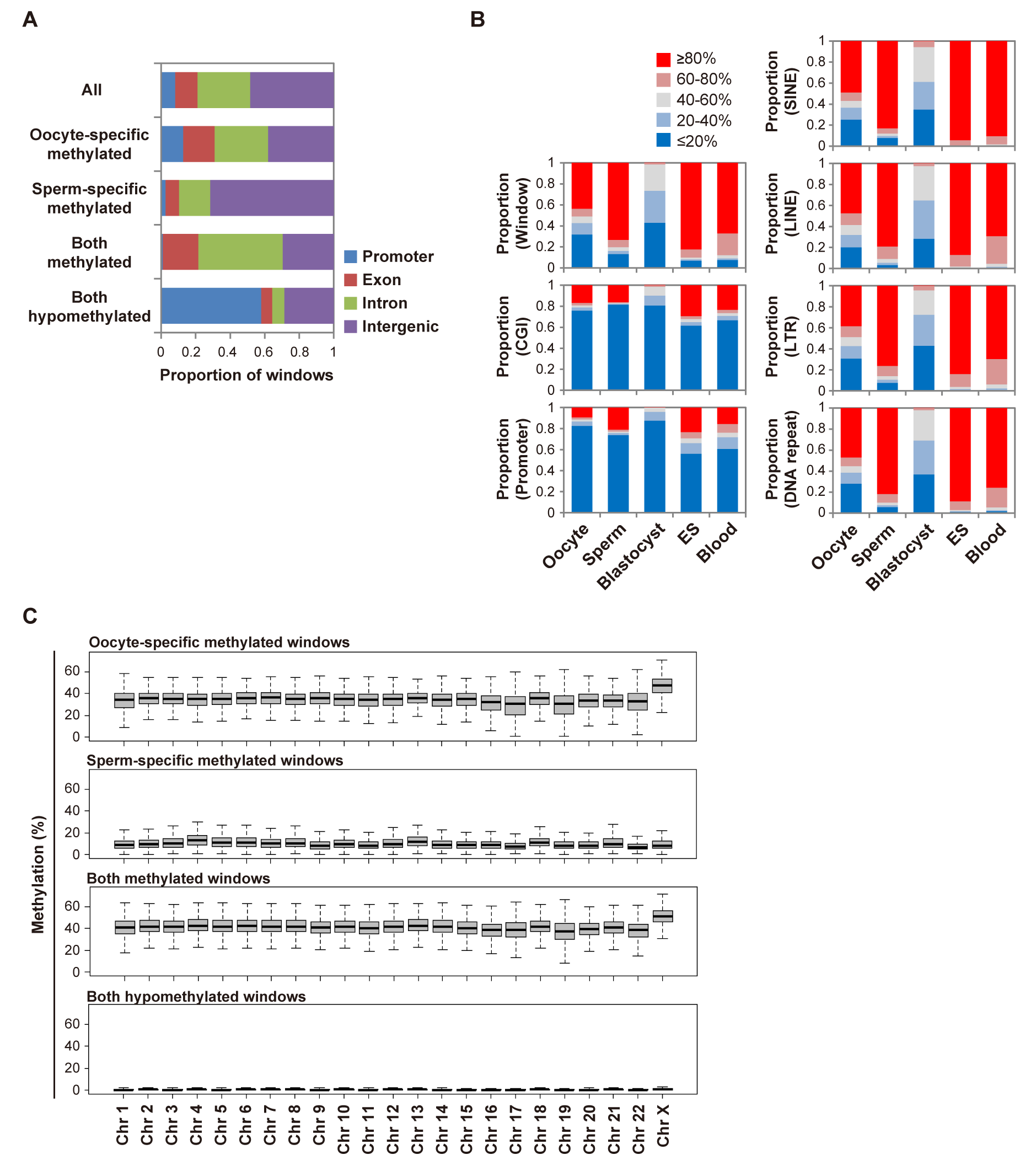

Supplement: S2 Figure — DNA methylation levels of specific genomic regions. A, Genomic distribution of windows. The proportions of windows overlapping promoters, exons, introns and intergenic regions are indicated. If a window overlaps more than two categories, the priority is as follows: 1) promoter, 2) exon, 3) intron, 4) intergenic region (e.g. if a window overlaps a promoter and an exon, it is classified as “promoter”). Sperm-specific methylated windows were abundant in intergenic regions. More than half of the windows hypomethylated in both gametes overlapped promoters. B, Distribution of mean methylation levels of windows, CGIs, promoters and repeat copies. A high proportion of hypomethylated repeat copies is evident in oocytes and blastocysts. C, Box plots of mean methylation levels of the sliding windows in human blastocysts. Boxes represent lower and upper quartiles and horizontal lines indicate the median. Whiskers extend to the most extreme data points within 1.5 times the interquartile range from the boxes. X-linked windows hypermethylated in oocytes showed ∼10% higher methylation levels than autosomal ones. (TIF) [file pgen.1004868.s002.tif]

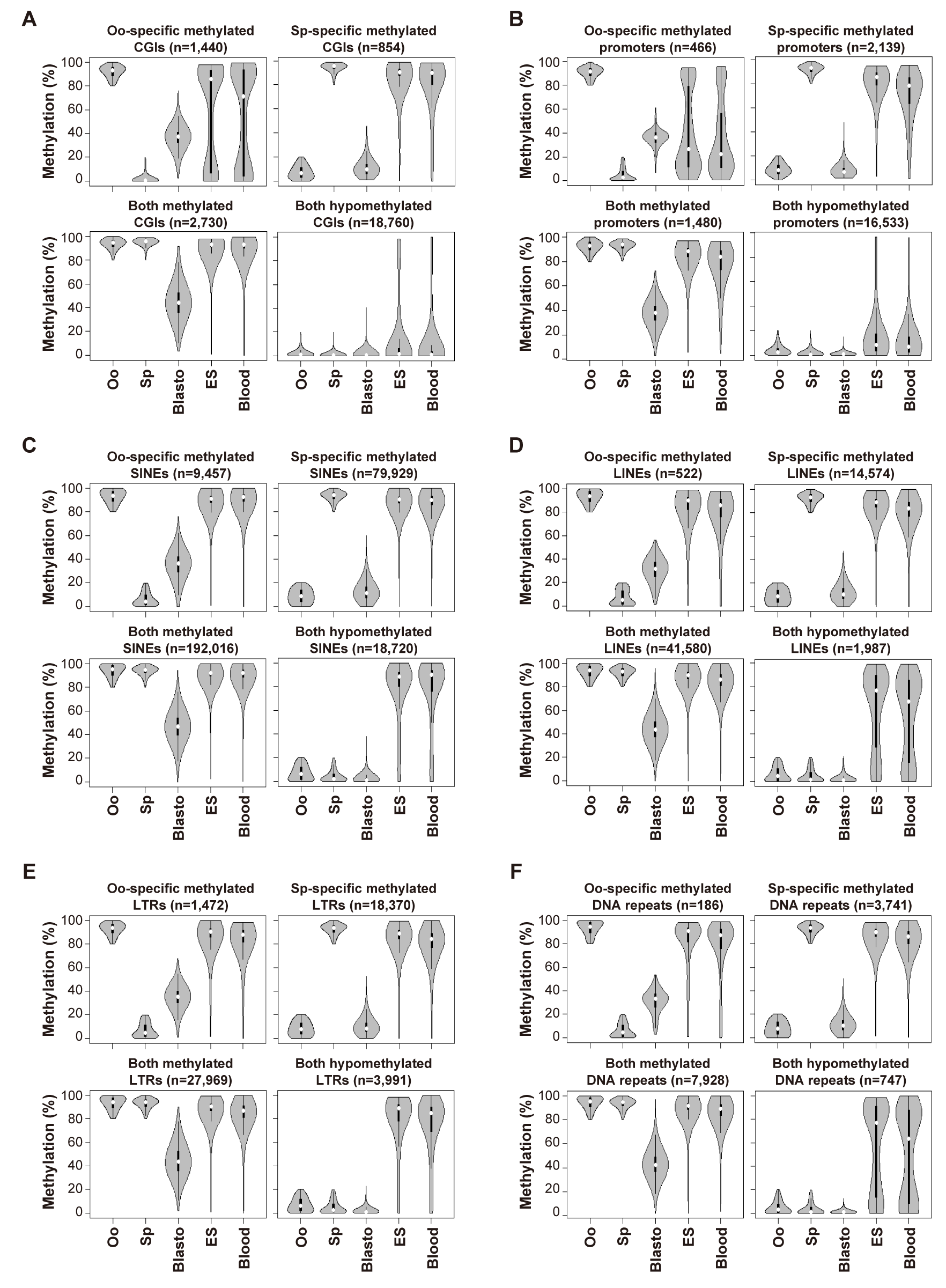

Supplement: S3 Figure — Region-specific methylation changes during early human development. A, Violin plots of mean methylation levels of CGIs. Thin and thick lines are box plots and white dots indicate the median. B, Violin plots of mean methylation levels of promoters. Oocyte-specific methylated promoters preferentially showed low methylation levels in ES and blood cells. C–F, Violin plots of mean methylation levels of repeat copies. SINEs, LINEs, LTRs and DNA repeats were demethylated similarly to other genomic regions in blastocysts. (TIF) [file pgen.1004868.s003.tif]

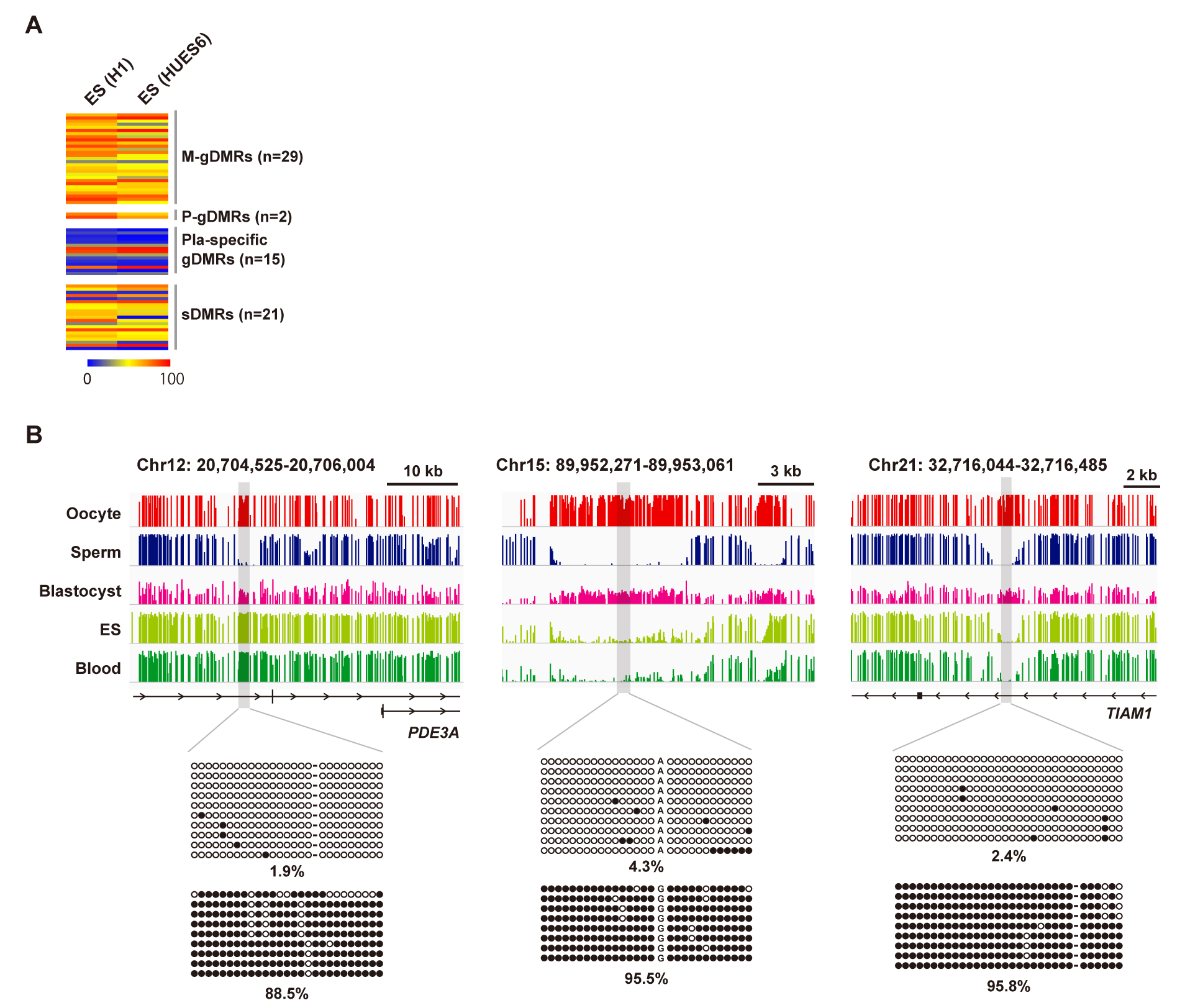

Supplement: S4 Figure — Stability of gDMRs and oocyte-specific methylated CGIs. A, A heatmap of mean methylation levels of gDMRs in H1 (GEO accession number: GSM429321) and HUES6 (GEO accession number: GSM1173778) ES cells. Among gDMRs other than placenta-specific ones, 13 and 9 DMRs showed>75% methylation in H1 and HUES6 ES cells, respectively. Methylation levels are color-coded as indicated. B, Methylation patterns of three oocyte-specific methylated CGIs. Black and white circles indicate methylated and unmethylated residues, respectively. The percentages of methylated CpG sites are indicated. (TIF) [file pgen.1004868.s004.tif]

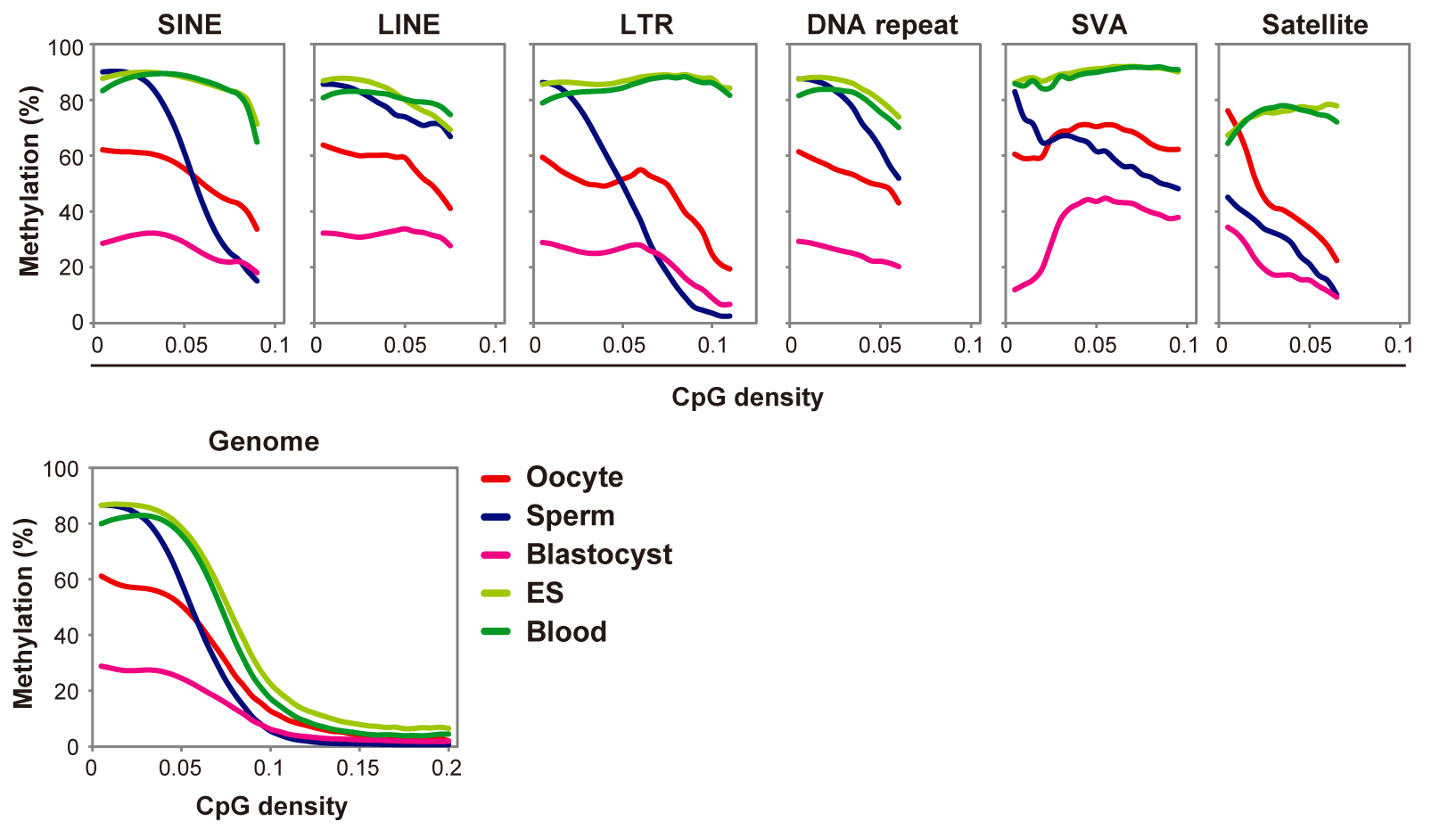

Supplement: S5 Figure — Relationships between methylation levels and CpG densities. Mean methylation levels of CpGs in six repeat families are plotted against CpG densities. All genomic CpGs were also analyzed for comparison. Mean methylation levels were calculated only for CpG densities with>1000 CpG sites covered by all samples. Transposable elements were essentially highly methylated in ES and blood cells. Low methylation levels of CpGs were observed in oocytes and blastocysts regardless of the CpG density. In sperm, CpGs in SINEs, LTRs and satellites showed especially low methylation levels at high CpG densities. (TIF) [file pgen.1004868.s005.tif]
